# Supplementary material for: Silicate dissolution boosts the CO2 concentrations in subduction fluids
Source: Nat Commun. 2017 Sep 20;8:616. doi: 10.1038/s41467-017-00562-z (PMC5606994; doi:10.1038/s41467-017-00562-z)
Supplement: Supplementary file 1 — Supplementary Information [file 41467_2017_562_MOESM1_ESM.pdf]

Description of Supplementary Files

File Name: Supplementary Information
Description: Supplementary Figures, Supplementary Tables and Supplementary References

File Name: Peer Review File

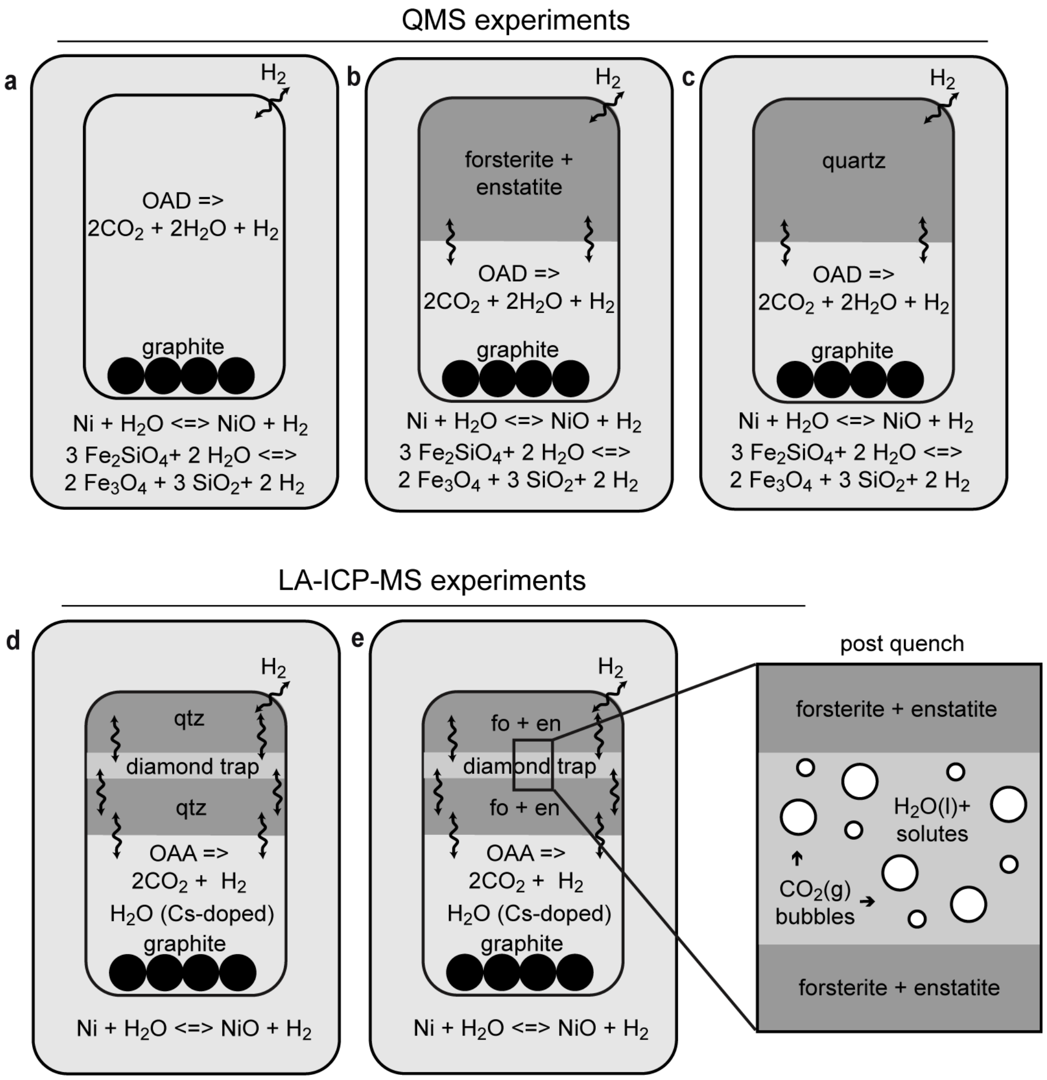

Supplementary figure 1: Experimental strategy adopted for the synthesis of COH fluids in the systems (a) COH; (b, d) MgO–SiO_2–COH and (c) SiO_2–COH. The fluid source is solid oxalic acid dihydrate (OAD), decomposing to carbon dioxide, water and hydrogen (molar ratio 2 : 2 : 1) at high-temperature conditions[1]. Figures a–c refer to experiments performed to determine volatile speciation using quadrupole mass spectrometry (QMS). Figures d–e refer to experiments performed for the analysis of dissolved SiO_2 and MgO using laser ablation ICP-MS (LA-ICP-MS). In all experiments, double capsules were employed, where fH_2 (and, consequently, fO_2) is buffered by either the assemblage Ni+NiO+H_2O (NNO) or Fe_2SiO_4+Fe_3O_4+SiO_2+H_2O (FMQ).

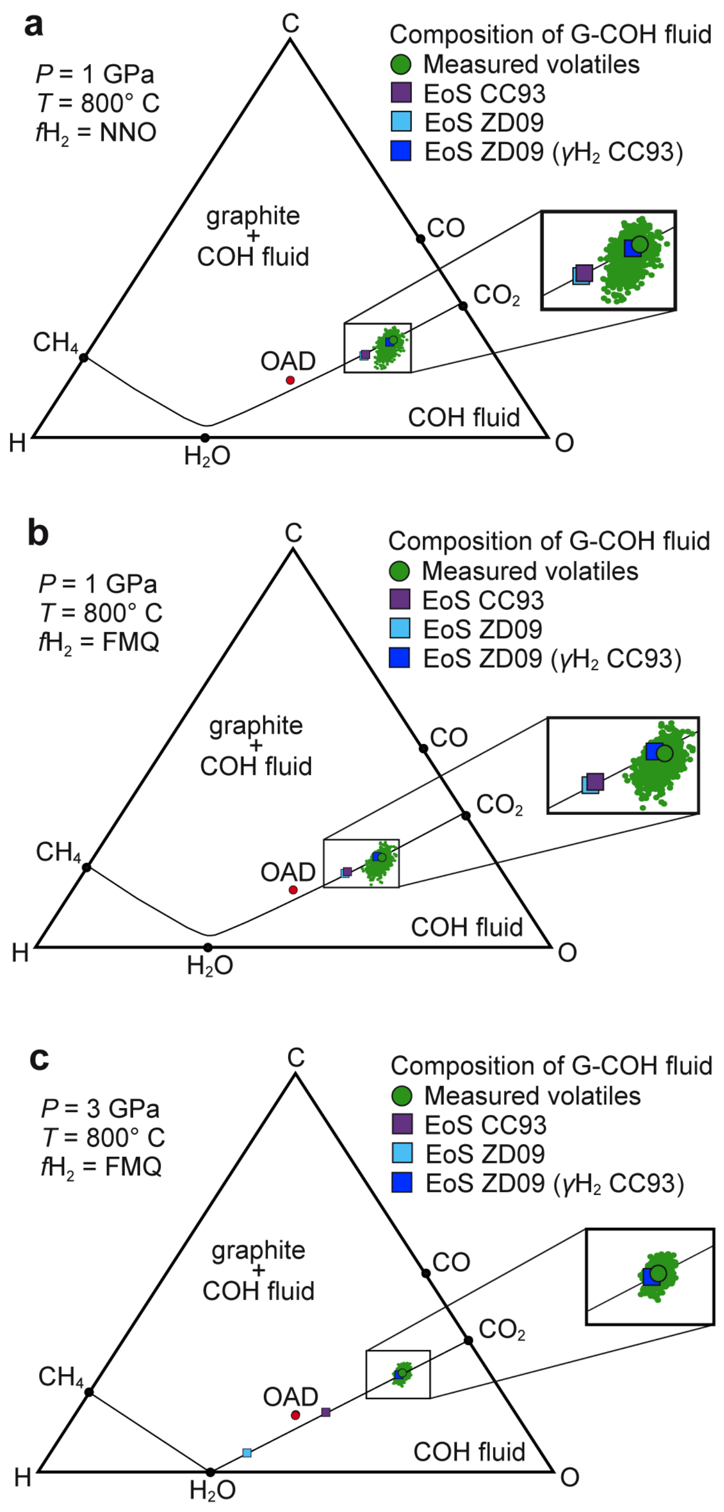

Supplementary figure 2: Ternary COH diagrams showing the composition of graphite-saturated and fH_2-buffered COH fluids at 1 GPa, 800° C (a, b) and 3 GPa, 800° C (c), measured by quadrupole mass spectrometry (large green dot: mean value; small green dots: analytical uncertainty) and estimated by thermodynamic modelling[2–4] [purple and blue squares; details on the equations of state (EoS) in supplementary table 1]. (a) experiments and calculations performed at fH_2^{NNO}; (b, c) experiments and calculations performed at fH_2^{FMQ}.

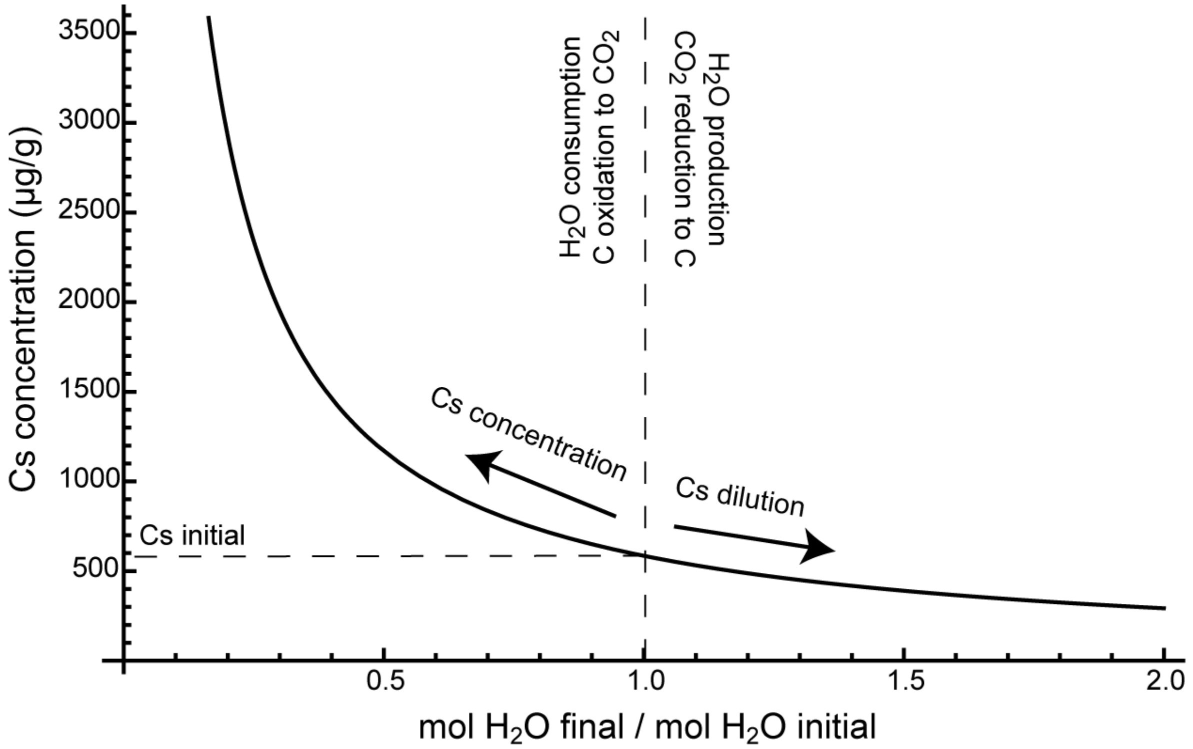

Supplementary figure 3: Variation of the Cs concentration in the water present in the inner capsule vs. the ratio between the initial moles of H_2O introduced in the capsule and the final moles of H_2O after re-equilibration at *P*, *T* and fH_2. The initial concentration of 590 µg g^{-1} increases in case of H_2 migration from the inner capsule to the outer capsule, with consequent H_2O consumption and graphite oxidation to CO_2.

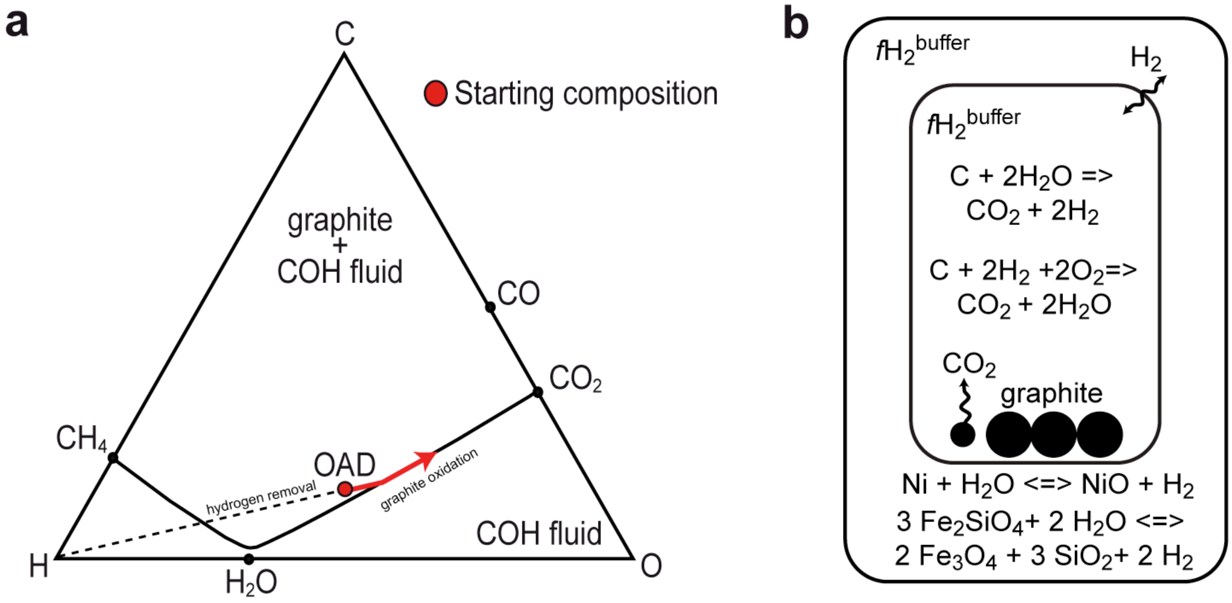

Supplementary figure 4: (a) Evolution of the COH fluid composition in the inner capsule at high *P*–*T* conditions (red arrow), starting from oxalic acid dihydrate (OAD) and ending in the measured final composition after equilibration with the fH_2 imposed by the buffer (NNO, FMQ) in the outer capsule. The graphite-saturation curve at 1 GPa and 800°C is shown for reference (black solid line). The increase of CO_2 in the fluid is accomplished by oxidation of graphite and hydrogen migration (red array) to the outer capsule through the H_2-permeable $Au_{60}Pd_{40}$ inner capsule, until the fH_2 in the inner capsule equals that in the outer capsule (b).

Supplementary table 1: Volatile composition of graphite-saturated COH fluids (mol%) in the systems COH, $MgO–SiO_2–COH$ and $SiO_2–COH$ measured by quadrupole mass spectrometry using the capsule-piercing technique. Estimated uncertainties are given in parentheses.

Run	COH70	CM10	FM20	COH69	CM8	FM19	COH76	COH77
System	COH	$MgO–SiO_2–$ COH	$SiO_2–COH$	COH	$MgO–SiO_2–$ COH	$SiO_2–COH$	COH	$SiO_2–COH$
P (GPa)	1	1	1	1	1	1	3	3
T (°C)	800	800	800	800	800	800	800	800
Redox buffer	NNO	NNO	NNO	FMQ	FMQ	FMQ	FMQ	FMQ
Runtime (*h*)	92	120	89	88	70	24	48	192
µmoles[a]	40.46	37.14	47.30	30.00	34.17	41.01	20.23	12.74
Measured volatiles (mol%)								
O_2	0.00	0.00	0.00	0.38(1.80)	0.24(2.11)	0.19(1.48)	0.00	0.56(1.31)
H_2O	26.91(0.97)	15.80(1.10)	17.16(0.92)	32.34(0.81)	11.25(0.96)	14.09(0.67)	25.56(0.49)	18.32(0.59)
H_2	0.25(0.20)	0.00	0.14(0.19)	0.00	0.00	0.00	0.00	0.00
CO	0.00	0.00	0.00	0.00	0.00	0.00	0.71(5.10)	0.00
CO_2	72.61(0.54)	84.20(0.61)	82.60(0.51)	66.99(0.45)	88.21(0.53)	85.53(0.37)	73.73(0.27)	81.12(0.33)
CH_4	0.22(0.88)	0.00	0.10(0.84)	0.28(0.74)	0.31(0.87)	0.20(0.61)	0.00	0.00
XCO_2[b]	0.73(0.01)	0.84(0.01)	0.83(0.01)	0.67(0.01)	0.89(0.01)	0.86(0.01)	0.74(0.01)	0.81(0.01)

[a]micromoles of gases evolved in the vessel, derived using the ideal gas law.

[b]$XCO_2 = CO_2 / (H_2O + CO_2)$

Supplementary table 2: Thermodynamic modeling of fH_2-buffered graphite-saturated COH fluids, simulating the double-capsule synthesis using the buffers NNO and FMQ.

Redox buffer	NNO			FMQ			FMQ		
P (GPa), *T* (° C)	1, 800			1, 800			3, 800		
log fO_2 outer capsule[a]	-13.47			-13.70			-11.19		
log fH_2 outer capsule = inner capsule[b]	1.775			1.889			2.361		
Modeled composition at fixed fH_2 (mol%)	CC93[2]	ZD09[3,c]	ZD09 mod[d]	CC93	ZD09	ZD09 mod	CC93	ZD09	ZD09 mod
H_2O	38.01	38.44	28.74	45.80	46.51	34.25	55.17	85.39	26.59
H_2	0.07	0.11	0.08	0.08	0.14	0.09	0.002	0.02	0.003
CO	0.5	0.62	0.66	0.43	0.57	0.64	0.02	0.03	0.07
CO_2	61.33	60.71	70.46	53.54	52.56	64.93	44.80	14.48	73.34
CH_4	0.09	0.13	0.06	0.15	0.22	0.09	0.001	0.07	0.001
XCO_2[e]	0.62	0.61	0.71	0.54	0.53	0.65	0.45	0.14	0.73
XO[f] inner capsule	0.678	0.674	0.746	0.624	0.618	0.705	0.568	0.401	0.765
log fO_2 inner capsule	-14.37	-14.34	-14.28	-14.43	-14.40	-14.31	-11.60	-12.36	-11.66
ΔFMQ[g] inner capsule	-0.67	-0.64	-0.58	-0.73	-0.70	-0.61	-0.42	-1.17	-0.47

[a]retrieved by modeling the reactions $2Ni+O_2 = 2NiO$ and $3Fe_2SiO_4 + O_2 = 2Fe_3O_4 + 3SiO_2$ using the Perple_X package[5] and the hp04ver.dat database.

[b]retrieved using Eq. 16 in the routine "fluids" in the Perple_X package (H-O HSMRK/MRK hybrid EoS).

[c]EoS by Zhang and Duan (2009)[3] with static H_2 fugacity coefficient (γH_2), changing as a function of *P*, *T* only (γH_2 = 5.440 at *P* = 1 GPa, *T* = 800° C; γH_2 = 112.0 at *P* = 3 GPa, *T* = 800° C).

[d]EoS by Zhang and Duan (2009)[3] with dynamic γH_2 by Connolly and Cesare (1993)[2], changing as a function of *P*, *T* and *X*O (γH_2 = 7.841 at *X*O = 0.746, *P* = 1 GPa, *T* = 800° C; γH_2 = 809.3 at *X*O = 0.765, *P* = 3 GPa, *T* = 800° C).

[e]$XCO_2 = CO_2 / (H_2O + CO_2)$

[f]$XO = O / (H + O)$

[g]$\Delta FMQ = \log fO_2 - \log fO_2^{FMQ}$.

Supplementary table 3: Solubility of forsterite + enstatite (systems MgO–SiO_2–COH and MgO–SiO_2–H_2O) and quartz (SiO_2–COH system) in experimental fluids at P = 1 GPa, T = 800° C and fH_2^{NNO} (run duration 48 h), measured by cryogenic laser-ablation ICP-MS and expressed as moles of SiO_2 and MgO per kilogram of water (mol kgH_2O^{-1}) and as weight percentage in the fluid (wt%). Uncertainties are given in parentheses as standard deviation of *n* measurements in the diamond trap. The concentration of the internal Cs standard, corrected taking into account the measured XCO_2 of the COH fluid in the MgO–SiO_2–COH and SiO_2–COH systems (supplementary figure 3), is also provided.

Run	CZ22	CZ29	CZ27
System	MgO–SiO_2–H_2O	MgO–SiO_2–COH	SiO_2–COH
P (GPa)	1	1	1
T (° C)	800	800	800
Redox buffer	n.a.	NNO	NNO
measured dissolved solutes (LA-ICP-MS)			
n	3	2	2
m SiO_2 (mol kgH_2O^{-1})	0.22 (0.06)	1.24 (0.19)	0.30 (0.04)
m MgO (mol kgH_2O^{-1})	0.28 (0.04)	1.08 (0.10)	n.a.
Total solutes (wt%)	2.4	11	1.8
Cs (μg g^{-1})	590	2140[a]	1370[b]

n.a. = not applicable

[a]initial Cs concentration (590 μg g^{-1}) corrected assuming XCO_2 = 0.84 (cf. run CM10, supplementary table 1).

[b]initial Cs concentration (590 μg g^{-1}) corrected assuming XCO_2 = 0.83 (cf. run FM20, supplementary table 1).

Supplementary table 4: Calculated model values of the solubility of forsterite + enstatite in water and forsterite + enstatite in COH fluid at fH_2^{NNO} compared with the experimentally measured values. Solubilities and aqueous species concentrations (molality). Experimental uncertainties in parentheses.

	Run	*P* (GPa)	*T* (°C)	log fH_2	pH	*m* SiO_2	*m* MgO	$Mg(OH)_2$[1]	MgSiC[2]	$Si(OH)_4$[3]	Si-dimer[4]
			forsterite + enstatite + graphite + COH fluid								
Exptl.	CZ29	1.0	800			1.24 (0.19)	1.08 (0.10)				
Calc.		1.0	800	1.78	3.73	1.15	1.22	0.11	1.11	0.039	0.00028
			forsterite + enstatite + H_2O								
Exptl.	CZ22	1.0	800			0.22 (0.06)	0.28 (0.04)				
Calc.		1.0	800	1.78	5.57	0.29	0.26	0.26	-	0.21	0.035

[1]$Mg(OH)_2$ stands for the molality of that species in the equilibrium $Mg(OH)_{2,aq} + 2H^+ = Mg^{2+} + 2H_2O$
for which the log *K* = 3.9 at 800°C and 1.0 GPa was obtained by fitting the experimental data for forsterite + enstatite + H_2O in the table.
[2]MgSiC stands for the molality of $Mg[OSi(OH_3)][CH_3CH_2COO]^0$ in the equilibrium $Mg[OSi(OH_3)][CH_3CH_2COO]^0 + H^+ = Mg^{2+} + SiO_{2,aq} + CH_3CH_2COO^- + H_2O$
for which the log *K* = -7.0 at 800°C and 1.0 GPa was obtained by fitting the experimental data for forsterite + enstatite + graphite + COH fluid in the table.
[3]$Si(OH)_4$ stands for the silica monomer molality.
[4]Si-dimer stands for the molality of $Si(OH)_3OSi(OH)_3$ in the equilibrium $Si(OH)_3OSi(OH)_3 + H_2O = 2Si(OH)_4$ for which the equilibrium constant log *K* = 0.1083 at 800°C and 1.0 GPa[6].

Supplementary table 5: H_2O and O_2 fugacities recalculated in order to fit fCO_2 retrieved from measurements (supplementary table 1), on the basis of Eq. 3, using fH_2 and fO_2 values from supplementary table 2 and fixed log K = 37.5 (see also Fig. 3).

Run	COH70	CM10	FM20	COH69	CM8	FM19
System	COH	MgO–SiO_2–COH	SiO_2–COH	COH	MgO–SiO_2–COH	SiO_2–COH
P (GPa)	1	1	1	1	1	1
T (°C)	800	800	800	800	800	800
Redox buffer	NNO	NNO	NNO	FMQ	FMQ	FMQ
recalculated fugacities						
fixed fH_2 and fO_2						
log fH_2O	3.628	3.596	3.600	3.708	3.648	3.655
fixed fH_2 and fH_2O						
log fO_2	-14.28	-14.25	-14.25	-14.31	-14.25	-14.26

Supplementary references:

1. Tiraboschi, C., Tumiati, S., Recchia, S., Miozzi, F. & Poli, S. Quantitative analysis of COH fluids synthesized at HP-HT conditions: an optimized methodology to measure volatiles in experimental capsules. *Geofluids* **16,** 841–855 (2016).
2. Connolly, J. a D. & Cesare, B. C-O-H-S Fluid Composition and Oxygen Fugacity in Graphitic Metapelites. *Journal of Metamorphic Geology* **11,** 379–388 (1993).
3. Zhang, C. & Duan, Z. A model for C-O-H fluid in the Earth's mantle. *Geochim. Cosmochim. Acta* **73,** 2089–2102 (2009).
4. Zhang, C. & Duan, Z. GFluid: An Excel spreadsheet for investigating C–O–H fluid composition under high temperatures and pressures. *Comput. Geosci.* **36,** 569–572 (2010).
5. Connolly, J. A. D. Computation of phase equilibria by linear programming: A tool for geodynamic modeling and its application to subduction zone decarbonation. *Earth Planet. Sci. Lett.* **236,** 524–541 (2005).
6. Sverjensky, D. A., Harrison, B. & Azzolini, D. Water in the deep Earth: The dielectric constant and the solubilities of quartz and corundum to 60kb and 1200°C. *Geochim. Cosmochim. Acta* **129,** 125–145 (2014).
